# Supplementary material for: Combining genomics and epidemiology to analyse bi-directional transmission of Mycobacterium bovis in a multi-host system
Source: eLife. 2019 Dec 17;8:e45833. doi: 10.7554/eLife.45833 (PMC6917503; doi:10.7554/eLife.45833)
Supplement: Transparent reporting form [file elife-45833-transrepform.docx]

***eLife’s* transparent reporting form**

We encourage authors to provide detailed information *within their submission* to facilitate the interpretation and replication of experiments. Authors can upload supporting documentation to indicate the use of appropriate reporting guidelines for health-related research (see [EQUATOR Network](http://www.equator-network.org/%20)), life science research (see the [BioSharing Information Resource](https://biosharing.org/)), or the [ARRIVE guidelines](http://www.plosbiology.org/article/info:doi/10.1371/journal.pbio.1000412) for reporting work involving animal research. Where applicable, authors should refer to any relevant reporting standards documents in this form.

If you have any questions, please consult our Journal Policies and/or contact us: [editorial@elifesciences.org](mailto:editorial@elifesciences.org).

**Sample-size estimation**

- You should state whether an appropriate sample size was computed when the study was being designed
- You should state the statistical method of sample size computation and any required assumptions
- If no explicit power analysis was used, you should describe how you decided what sample (replicate) size (number) to use

Please outline where this information can be found within the submission (e.g., sections or figure legends), or explain why this information doesn’t apply to your submission:

The *Mycobacterium bovis* isolates used for the current analyses were selected retrospectively from archives. Sample size calculations weren’t conducted because the number of samples was restricted to those available in the archives and there was a lack of sufficient data regarding the prevalence of infection in the underlying populations. Where selection was conducted, isolates were selected in an attempt to create a representative sample of the *M. bovis* population circulating in the badger and cattle populations within and surrounding Woodchester Park. Full details regarding the isolate selection are available in the *Methods: Selecting the Isolates* section.

**Replicates**

- You should report how often each experiment was performed
- You should include a definition of biological versus technical replication
- The data obtained should be provided and sufficient information should be provided to indicate the number of independent biological and/or technical replicates
- If you encountered any outliers, you should describe how these were handled
- Criteria for exclusion/inclusion of data should be clearly stated
- High-throughput sequence data should be uploaded before submission, with a private link for reviewers provided (these are available from both GEO and ArrayExpress)

Please outline where this information can be found within the submission (e.g., sections or figure legends), or explain why this information doesn’t apply to your submission:

A single *M. bovis* isolate was considered for each sampled badger and cow associated with the current research. Therefore, each isolate should represent an independent sample of the *M. bovis* population. Some *M. bovis* sequences were excluded due to issues with quality and metadata inconsistencies. The number of sequences excluded and the reasons for doing so are detailed in the *Methods: Generating and processing the sequencing data* section. In addition, this information is summarized in *Supplementary Figure 1*. The sequence data generated in the current research has been uploaded to the NCBI SRA (National Center for Biotechnology Information Sequence Read Archive) database under the bioproject accession number: PRJNA523164 (<https://www.ncbi.nlm.nih.gov/bioproject/PRJNA523164>). These data will be released when the manuscript is published.

**Statistical reporting**

- Statistical analysis methods should be described and justified
- Raw data should be presented in figures whenever informative to do so (typically when N per group is less than 10)
- For each experiment, you should identify the statistical tests used, exact values of N, definitions of center, methods of multiple test correction, and dispersion and precision measures (e.g., mean, median, SD, SEM, confidence intervals; and, for the major substantive results, a measure of effect size (e.g., Pearson's r, Cohen's d)
- Report exact p-values wherever possible alongside the summary statistics and 95% confidence intervals. These should be reported for all key questions and not only when the p-value is less than 0.05.

Please outline where this information can be found within the submission (e.g., sections or figure legends), or explain why this information doesn’t apply to your submission:

The statistical analyses used are described in detail in the *Methods* sections: *Comparing genetic and epidemiological distances*, *Building phylogeny and interrogating clusters*, and *Estimating inter-species transmission rates*. The appropriateness of the statistical analyses is discussed in the *Discussion* section. Further details describing the statistical analyses used are provided in the following *Supplementary Information* sections: *Using epidemiological metrics in Random Forest* *analyses* and *Phylogenetic analyses*.

(For large datasets, or papers with a very large number of statistical tests, you may upload a single table file with tests, Ns, etc., with reference to sections in the manuscript.)

**Group allocation**

- Indicate how samples were allocated into experimental groups (in the case of clinical studies, please specify allocation to treatment method); if randomization was used, please also state if restricted randomization was applied
- Indicate if masking was used during group allocation, data collection and/or data analysis

Please outline where this information can be found within the submission (e.g., sections or figure legends), or explain why this information doesn’t apply to your submission:

The *M. bovis* isolates analysed here were in predetermined groups based on the species they were derived from, badgers or cattle. The numbers of isolates sourced from badgers and cattle is described in the *Methods: Selecting the Isolates* and *Generating and processing the sequencing data* sections. For the phylogenetic analyses, further groups were defined using the sampling locations associated with each *M. bovis* isolate, details are provided in the *Methods: Estimating inter-species transmission rates* section.

**Additional data files (“source data”)**

- We encourage you to upload relevant additional data files, such as numerical data that are represented as a graph in a figure, or as a summary table
- Where provided, these should be in the most useful format, and they can be uploaded as “Source data” files linked to a main figure or table
- Include model definition files including the full list of parameters used
- Include code used for data analysis (e.g., R, MatLab)
- Avoid stating that data files are “available upon request”

Please indicate the figures or tables for which source data files have been provided:

Figure 1 – The sequencing data used to generate this phylogenetic tree have been uploaded to the NCBI SRA database under bioproject accession number: PRJNA523164.

Unfortunately, the badger and cattle population databases - managed by the Animal & Plant Health Agency (APHA) - cannot be made publicly available due to their sensitive nature. As a result, most of the source data used to create the figures in the current manuscript cannot be made available.

All the code used to create the figures and conduct the underlying analyses are freely available on github: <https://github.com/JosephCrispell/GeneralTools/tree/master/WoodchesterPark>.
